# Phase I trial comparing bile acid and short-chain fatty acid alterations in stool collected from human subjects treated with omadacycline or vancomycin

**DOI:** 10.1128/aac.01251-24

**Published:** 2025-01-17

**Authors:** Jinhee Jo, Chenlin Hu, Thomas D. Horvath, Sigmund J. Haidacher, Khurshida Begum, M. Jahangir Alam, Kevin W. Garey

**Affiliations:** 1 Department of Pharmacy Practice and Translational Research, University of Houston College of Pharmacy 15507 , Houston, Texas, USA; 2 Department of Pathology and Immunology, Baylor College of Medicine 3989 , Houston, Texas, USA; 3 Texas Children’s Microbiome Center, Department of Pathology, Texas Children’s Hospital 649289 , Houston, Texas, USA; University of California, San Francisco, San Francisco, California, USA

**Keywords:** *Clostridioides difficile*, human subjects, bile acids, short-chain fatty acids

## Abstract

Omadacycline, an aminomethylcycline tetracycline, has a low propensity to cause *Clostridioides difficile* infection (CDI) in clinical trials. Omadacycline exhibited a reduced bactericidal effect compared with vancomycin on key microorganisms implicated in bile acid homeostasis and short-chain fatty acids (SCFAs), key components of CDI pathogenesis. The purpose of this study was to assess bile acid and SCFA changes in stool samples from healthy volunteers given omadacycline or vancomycin. Stool samples were collected daily from 16 healthy volunteers, who were given oral omadacycline or vancomycin for 10 days. Daily stool samples were assessed for bile acids and SCFA concentrations using liquid chromatography-tandem mass spectrometry (LC-MS/MS). Bile acids changed significantly over time for all subjects (*P* < 0.01 for each bile acid), with vancomycin causing a larger change in the primary bile acids, cholic acid (*P* < 0.001) and chenodeoxycholic acid (*P* < 0.001), and a reduced change in the secondary bile acid, lithocholic acid (*P* < 0.001). The secondary bile acid ursodeoxycholic acid was reduced less by vancomycin than by omadacycline (*P* < 0.001). All SCFA concentrations were reduced from baseline with a larger effect observed with vancomycin for isobutyric acid (*P* = 0.0034), propionic acid (*P* = 0.0012), and acetic acid (*P* = 0.047). Microbial changes associated with the use of vancomycin versus omadacycline were also associated with changes in bile acid homeostasis and SCFA concentrations. Oral omadacycline produced a distinctive metabolomic profile compared with vancomycin when administered to healthy subjects. The metabolic findings help further our understanding of the lower CDI risk properties of omadacycline and warrant phase 2 investigations using omadacycline as a CDI antibiotic.

**IMPORTANCE:**

The purpose of this study was to assess bile acid and SCFA changes in stool samples obtained from healthy volunteers given omadacycline or vancomycin. Stool samples were collected daily from 16 healthy volunteers given a 10-day oral course of omadacycline or vancomycin. Vancomycin caused a larger change in the primary bile acids and SCFA concentrations compared with omadacycline. The metabolic findings help further our understanding of the mechanistic basis for the lower-risk properties of omadacycline causing CDI and warrant phase 2 investigations using omadacycline as a CDI antibiotic.

**CLINICAL TRIALS:**

This study is registered with ClinicalTrials.gov as NCT06030219.

## INTRODUCTION

*Clostridioides difficile* infection (CDI) is associated with high incidence, mortality, and healthcare-related costs (1–3). Patients with CDI suffer from high recurrence rates of approximately 20%–25% following an initial infection and increasing to 50% in recurrent cases (4). The development of CDI is characterized by the disruption of the human gut microbiota, most often due to the use of broad-spectrum antibiotics. Thus, an ideal therapeutic agent for treating CDI should exhibit minimal impact on the gut microbiota while possessing potent activity against *C. difficile*. Oral vancomycin, the most commonly used antibiotic for CDI treatment, not only has potent activity against *C. difficile* but also has broad-spectrum activity against the gut microbiota and high recurrence rates (5, 6). Also, there are increasing reports of antibiotic resistance development against the currently approved agents, metronidazole, vancomycin, and fidaxomicin (7). Thus, there is a pressing need for the development of antimicrobial compounds with targeted activity against *C. difficile* while preserving the beneficial gut microbiota and its functions.

Omadacycline, an aminomethylcycline tetracycline antibiotic, has demonstrated a potent activity against *C. difficile* and a low propensity to cause antibiotic-induced CDI in clinical trials (8–10). We recently completed a phase I healthy volunteer study comparing microbiome changes in adults given oral omadacycline or oral vancomycin (11). Notable metagenomic differences were observed in subjects given omadacycline including the preservation of key phyla Bacillota and Actinomycetota. These phyla have important biological roles in bile acid homeostasis and the fermentation of dietary fiber into short-chain fatty acids (SCFAs). Given these metagenomic differences, we hypothesized that microbiome-based metabolic processes would also be affected. The purpose of this study was to perform targeted metabolomic analysis on stool samples obtained from healthy volunteers given omadacycline or vancomycin. The results from this study should help elucidate the mechanism underlying omadacycline’s low risk for CDI and provide a better understanding of gut microbial-based metabolic changes associated with omadacycline and vancomycin.

## MATERIALS AND METHODS

### Materials

Standards for primary bile acids, cholic acid (CA) and chenodeoxycholic acid (CDCA), and secondary bile acids, lithocholic acid (LCA), deoxycholic acid (DCA), and ursodeoxycholic acid (UDCA), were purchased from Sigma-Aldrich (St. Louis, MO). Optima liquid chromatography-tandem mass spectrometry (LC-MS)-grade solvents including water, acetonitrile, and methanol, and LC-MS-grade formic acid were all purchased from Fisher Scientific (Waltham, MA, USA) for the SCFA method. The derivatizing reagent, 1-(3- dimethylaminopropyl)−3-ethylcarbodiimide hydrochloride (EDAC), and the quenching reagents, succinic acid and 2-mercaptoethanol, were all purchased from Fisher Scientific. Unlabeled aniline (used in the synthesis of the unlabeled SCFA standards) and [^13^C_6_]-aniline (used in the synthesis of SCFA-based internal standard compounds) were purchased from Millipore-Sigma (Burlington, MA, USA). Standards for butyric acid, isobutyric acid, 2-methylbutyric acid, acetic acid, propionic acid, formic acid, valeric acid, isovaleric acid, and hexanoic acid were purchased from Millipore-Sigma.

### Description of samples and metagenomic analysis

Stool samples were collected daily as part of our phase 1, non-blinded, randomized clinical trial with healthy volunteers study receiving either oral omadacycline or vancomycin (ClinicalTrials.gov number NCT06030219) (11). Briefly, healthy volunteers aged 18–40 years received a 10-day course of antibiotics, and stool samples were collected at baseline (day 0), daily during therapy (days 1–10), and two follow-up visits (days 13 and 30). All samples were processed immediately following collection at the central laboratory located in the University of Houston College of Pharmacy and stored at −80°C until further analyses. For metagenomic analysis, stool DNA was extracted using the MagAttract Power Microbiome Kit. The V4 region of the 16S RNA gene was sequenced using an Illumina MiSeq as previously described (11).

### Bile acid extraction and analysis

Stool samples were weighed, aliquoted, and solubilized with a 10 µL volume of methanol per 1 mg of stool. The mixture was then incubated overnight at 4°C, underwent ultrasonification for 5 min, and centrifuged at 10,000 g for 3 min. The supernatants from all samples were transferred to a new tube and analyzed using LC-MS/MS for the analysis of unconjugated primary (CA and CDCA) and secondary (DCA, LCA, and UDCA) bile acids as previously described (12). The final concentration of each bile acid was normalized by the corresponding stool sample weight.

### Short-chain fatty acid extraction and analysis

Stool samples were aliquoted with fecal weights ranging from 50 to 100 mg for each sample. Each stool aliquot was placed on ice with a 200 µL volume of methanol. After centrifuging, the supernatant was collected into a new tube, and the stool sample extracts were stored frozen at −80°C until the SCFA derivatization procedure could be performed. Prior to derivatization, each stool sample extract was thawed at ambient temperature on the laboratory benchtop. In a fresh tube, a 35 µL volume of each fecal sample extract was diluted with a 35 µL volume of acetonitrile, and the tubes were capped and vortex-mixed for 20 s. Then, a 20 µL volume of an unlabeled aniline solution (100 mM) and a 10 µL volume of an EDAC solution (100 mM) were added to each sample tube followed by vortex-mixing for 20 s, and the samples were then incubated at 4°C for 2 h. After completion of the derivatization, the reaction in each sample was quenched by the addition of a 12 µL volume of a 2-mercaptoethanol solution (100 mM) and an 18.8 µL volume of succinic acid solution (250 mM), followed by vortex-mixing for 20 s, and the samples were incubated at 4°C for 2 h. Prior to performing the SCFA-based bioanalysis by LC-MS/MS as previously described (13, 14), the samples were diluted an additional 10-fold in an internal standard solution yielding an overall dilution factor of 37.4. The final concentration of each SCFA was normalized by the corresponding stool sample weight.

### Statistical analysis

SAS version 9.1 was used for all statistical analyses. Baseline differences in bile acid and SCFAs were assessed by regression analysis, with demographic variables assessed using multivariable modes. The changes in bile acids and SCFAs over time compared with baseline in response to omadacycline or vancomycin were analyzed using two-way factorial design analysis of variance (ANOVA) with Duncan test for repeated measures. The effect of omadacycline and vancomycin on microbiome composition at the order level, controlling for multiple sample days per subject, was assessed using MaAslin2 (15). MaAsLin2 allows for multivariable associations between metabolomics and microbiota changes or antibiotics assigned using general linear models, accommodating longitudinal samples over time. For any bile acid or SCFA that differed significantly between vancomycin and omadacycline, microbiome composition was compared between subject samples that did or did not have changes in bile acids or SCFAs using MaAslin2. For bile acid analysis, subject samples were compared between those who did or did not have decreased concentration of each bile acid compared with baseline. As all patients had decreased SCFA concentrations, subjects were compared based on a ±75% decrease in each SCFA from baseline. Data visualization was performed using R (16).

## RESULTS

### Study design

A total of 16 healthy subjects aged between 18 and 40 years were recruited between 2020 and 2021 and received a 10-day course of either oral omadacycline or vancomycin. A comprehensive description of this phase I study including safety, fecal pharmacokinetics, and initial gut microbiome effects has been previously published (11). A total of 162 stool samples collected at baseline (day 0) and daily during antibiotic therapy (days 1–10) were used for bile acid and SCFA analyses.

### Bile acids

**Fig 1:**
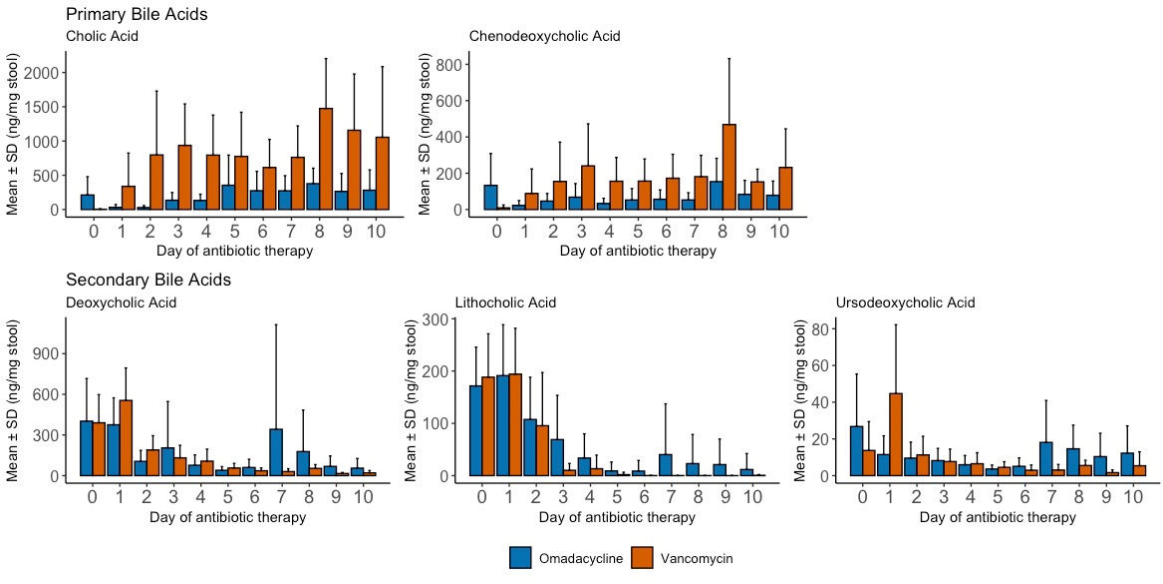
Bile acid changes over time in healthy subjects given a 10-day course of oral omadacycline or oral vancomycin.

There was no significant difference between total primary bile acids (*P* = 0.0523) or total secondary bile acids (*P* = 0.96) at baseline. Baseline primary bile acids were more variable for subjects given omadacycline (range: 7.7–1268 ng/mg) than for those given vancomycin (range: 1.6–64 ng/mg). Individually, baseline cholic acid concentrations were significantly higher in subjects assigned to omadacycline (213 ± 267 ng/mg) compared with vancomycin (4.6 ± 7.8 ng/mg; *P* = 0.045). Other individual primary and secondary acids did not differ between the groups. The concentration changes over time of primary and secondary bile acids are shown in Fig. 1. All five bile acids (cholic, chenodeoxycholic, deoxycholic, lithocholic, and ursodeoxycholic acids) changed significantly over time in subjects given omadacycline or vancomycin (*P* < 0.01 for each bile acid). For primary bile acids, the average daily change in cholic acid from baseline was significantly higher in vancomycin-treated subjects (+712.2 ng/mg) compared with omadacycline-treated subjects (−0.98 ng/mg; *P* < 0.001). Chenodeoxycholic acid average daily change from baseline was also significantly higher in vancomycin-treated subjects (+142.3 ng/mg) compared with the decreased concentrations observed in omadacycline-treated subjects (−70.7 ng/mg; *P* < 0.001). For secondary bile acids, the average daily change in deoxycholic acid was similar in vancomycin-treated subjects (−253.8 ng/mg) and omadacycline-treated subjects (−186.0 ng/mg; *P* < 0.001). Lithocholic acid decreased more in vancomycin-treated subjects (−138.0 ng/mg) compared with omadacycline-treated subjects (−104.7 ng/mg; *P* < 0.001). The average daily change from baseline in ursodeoxycholic acid decreased less in vancomycin-treated subjects (−4.7 ng/mg) compared with omadacycline-treated subjects (−12.6 ng/mg; *P* < 0.001). In omadacycline-treat subjects, secondary bile acid concentrations initially declined and then increased after approximately day 7 of antibiotic therapy, which was unique to omadacycline compared with vancomycin.

### Short-chain fatty acids

**Fig 2:**
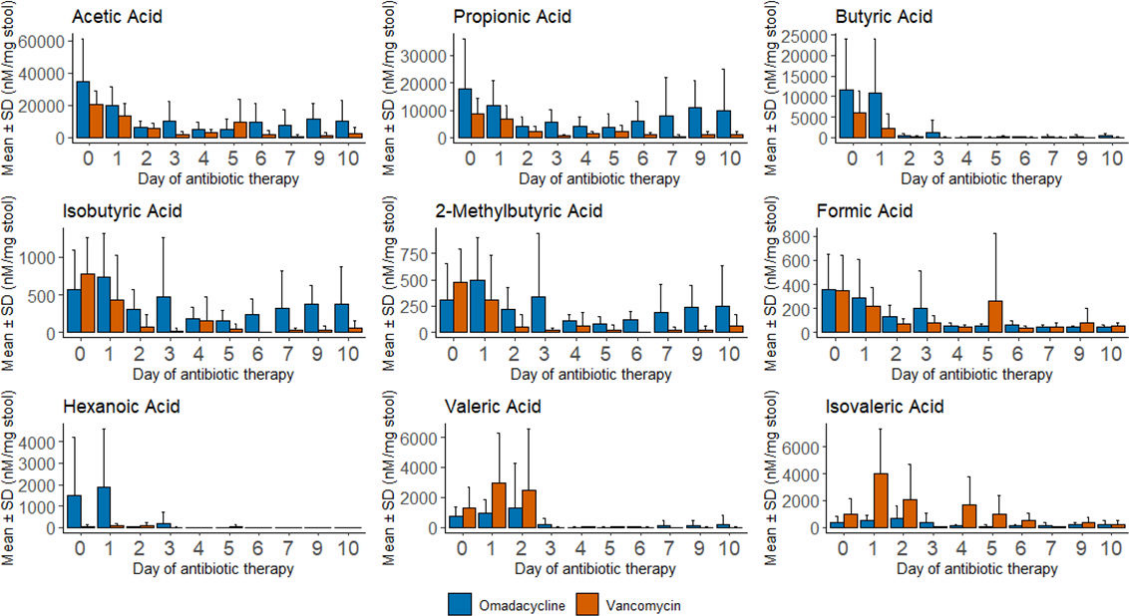
Short-chain fatty acid changes over time in healthy subjects given a 10-day course of oral omadacycline or oral vancomycin.

There were no differences at baseline in SCFA concentrations between subjects given omadacycline or vancomycin (Fig. 2). Percent change in SCFA from baseline is shown in Table S1. Using two-way ANOVA, changes in SCFAs from baseline over time were significant for acetic acid, propionic acid, butyric acid, isovaleric acid, and valeric acid, regardless of treatment. Significantly reduced percent change from baseline for isobutyric acid (−9% vs −74%; *P* = 0.0034), propionic acid (−40% vs −65%; *P* = 0.0012), and acetic acid (−9% vs −66%; *P* = 0.047) was observed in omadacycline-treated subjects compared with vancomycin-treated subjects. For these SCFAs, omadacycline-treated subjects initially had declined SCFA concentrations from baseline, which increased after approximately day 6 of antibiotic therapy. This rebound in SCFAs was not observed in vancomycin-treated subjects.

### Microbial and metabolomic changes

**Fig 3:**
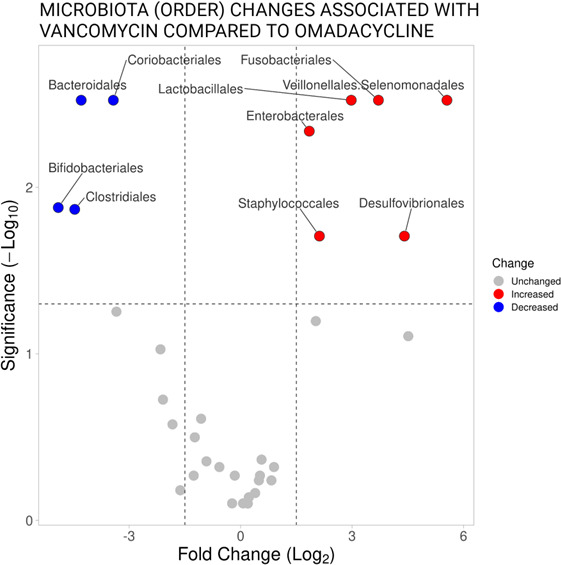
Microbiota taxa associated with relative abundance changes in subjects given vancomycin using subjects given omadacycline as a comparator.

Using MaAsLin2, omadacycline treatment was associated with a higher proportion of Bacteroidales, Coriobacteriales, Bifidobacteriales, and Clostridiales orders and lower concentrations of Staphylococcales, Fusobacteriales, Lactobacillales, Veillonellales, Selenomonades, Enterobacterales, and Desulfovibrionales orders (Fig. 3). Microbial changes associated with cholic acid, chenodeoxycholic acid, and lithocholic acid were analyzed using MaAsLin2 (Fig. S1). Enterobacterales were most highly associated with increased cholic acid and decreased lithocholic acid. Coriobacteriales and Bifidobacteriales were associated with decreased cholic acid and increased lithocholic acid. Microbial changes associated with acetic acid, propionic acid, and isobutyric acid are also shown in Fig. S1. Similar to the bile acid analysis, changes in the microbiota that were associated with the receipt of omadacycline or vancomycin were also associated with changes in SCFAs.

## DISCUSSION

Omadacycline, an aminomethylcycline tetracycline antibiotic, has a low propensity to cause CDI despite its high colonic concentrations and broad activity against aerobic and anaerobic bacteria including *C. difficile*. In our recently published microbiome paper (11), healthy subjects given omadacycline exhibited a distinctly different gut microbiome profile compared with subjects given vancomycin, as measured by Bray-Curtis dissimilarity index. This was primarily due to different effects on the gut microbiota with metabolic functions including known producers of SCFAs and biotransformers of primary to secondary bile acids. These microbial metabolic functions have important implications in the pathogenesis of CDI (17, 18). Using samples from our phase 1, healthy volunteers study, the purpose of this study was to characterize bile acid and SCFA differences in healthy subjects to further understand the anti-CDI properties of omadacycline compared with vancomycin. The results from this study demonstrated that omadacycline caused less disruption to bile acid homeostasis and less effect on certain SCFAs. We are able to directly correlate changes in the microbiome to these metabolic changes. Strengths of the study include the collection of samples during a rigorous phase I study design, parallel assessment of two known metabolites associated with CDI, daily assessment allowing for evaluation of fluctuation of changes during treatment, and confirmation of microbial changes shown previously using a new analysis technique, MaAsLin2.

### Bile acid and short-chain fatty acid changes and CDI

The role of bile acid homeostasis and CDI is evolving. Anaerobic bacteria residing in the gastrointestinal (GI) tract are largely responsible for converting primary bile acids to secondary bile acids via 7-α-dehydroxylation. Only a relatively small group of commensal bacteria can perform 7-α-dehydroxylation, including Lachnospiraceae and Ruminococcaceae family members belonging to Clostridiales order (19). These bacterial orders were less affected by omadacycline compared with vancomycin, confirming the importance of these organisms in bile acid homeostasis. This also shows, for the first time, the protective effect of omadacycline on bile acid homeostasis. The results of the MaAsLin2 analysis identified Enterobacterales as a key predictor for either an increase in the primary bile acid, cholic acid, or a decrease in the secondary bile acid, lithocholic acid. Bacterial taxa contributing to bile acid metabolism are varied but generally do not include Enterobacterales (20). Enterobacterales (Gammaproteobacteria) has been shown to be a marker of microbiome dysbiosis in other studies, and we hypothesize that the identification of Enterobacterales is likely a surrogate marker for the loss of multiple other species involved in bile acid metabolism (21).

The role of SCFA in CDI is also evolving but less clear. Commensal bacteria are responsible for producing SCFAs via anaerobic fermentation of non-digestible, complex carbohydrates (22). Acetate, butyrate, and propionate are the most abundant types of SCFAs in the GI tract with an approximate ratio of 60:20:20, accounting for nearly 90% of SCFAs in the GI tract (23, 24). A similar ratio was observed at baseline in our healthy volunteers with quite a large variation in SCFA concentrations between subjects. SCFA-producing bacteria include members belonging to Bifidobacteriales and Coriobacteriales orders (25). Acetate has recently been shown to have a protective role in gut microbiota disrupted by *C. difficile* (26), and butyrate has been shown to protect against CDI in mice via bile acid metabolism regulation (27). Subjects given omadacycline had significantly less disruption in acetate and propionate production compared with those given vancomycin. Like bile acids, these SCFAs were initially depleted in omadacycline-treated subjects but increased after approximately day 6 of antibiotic therapy. Butyrate was similarly depleted in subjects given omadacycline or vancomycin, but a branched chain SCFA, isobutyric acid, was significantly less depleted due to increased concentrations observed after day 6 of antibiotic therapy. These results will need to be confirmed in patients with infectious diseases including CDI, but they provide a better mechanistic understanding of how omadacycline is considered a low-risk antibiotic to cause CDI and may be less likely to cause CDI recurrence if used for the treatment of CDI.

Our study has several limitations. In this phase 1 study, we only enrolled eight subjects per treatment group. Although we were able to identify statistically significant associations, we were not powered to be able to perform more detailed analyses. Our microbiome evaluation used 16S rRNA sequencing; thus, we were not able to perform bacterial species or strain-level analyses. These results will need to be expanded with a deeper sequencing method to perform further correlation analyses. We performed targeted metabolomics on bile acids and SCFAs, two metabolic pathways implicated in the pathogenesis of CDI. However, these analyses limit the identification of unique metabolic mechanisms that explain the low propensity of omadacycline to cause CDI. Additionally, our short study period limits analyses on time to normalization of bile acids and SCFAs after discontinuation of antibiotics. Per-subject data are quite heterogeneous and non-linear. We used MaAslin2 to help account for our multiple-day sampling strategy; however, more advanced techniques to analyze these data types are needed. Finally, our study involved healthy volunteers without preexisting comorbidities; thus, an evaluation of omadacycline in patients with CDI or other infectious diseases is warranted.

### Conclusions

Oral omadacycline produced a distinctive metabolomic profile compared with vancomycin when administered to healthy subjects. The metabolic findings help further our understanding of the mechanistic basis for the low-risk properties of omadacycline in causing CDI and warrant phase 2 investigations using omadacycline as a CDI antibiotic

## SUPPLEMENTAL MATERIAL

Figure S1 aac.01251-24-s0001.png Bacterial taxon (order) changes associated with bile acid and short-chain fatty acids.

Table S1 aac.01251-24-s0002.docx Short-chain fatty acid proportional change from baseline in subjects given omadacycline compared to vancomycin.

ASM does not own the copyrights to Supplemental Material that may be linked to, or accessed through, an article. The authors have granted ASM a non-exclusive, world-wide license to publish the Supplemental Material files. Please contact the corresponding author directly for reuse.
