# Supplementary figures and images for: Phase I trial comparing bile acid and short-chain fatty acid alterations in stool collected from human subjects treated with omadacycline or vancomycin
Source: Antimicrob Agents Chemother. 2025 Jan 17;69(2):e01251-24. doi: 10.1128/aac.01251-24 (PMC11823362; doi:10.1128/aac.01251-24)

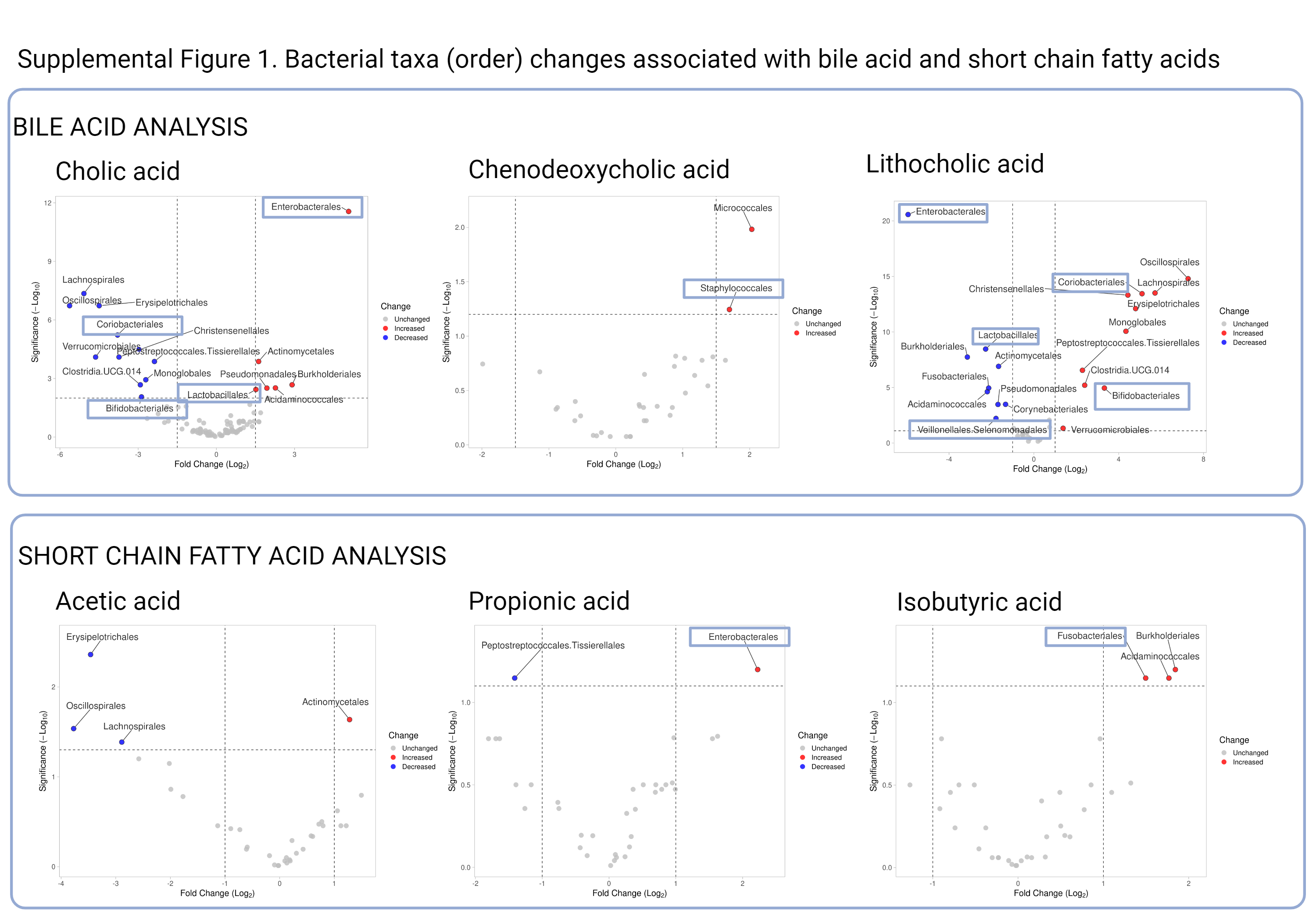

Supplement: Figure S1 — Bacterial taxon (order) changes associated with bile acid and short-chain fatty acids. [file aac.01251-24-s0001.png]
