# Supplementary material for: Phase I trial comparing bile acid and short-chain fatty acid alterations in stool collected from human subjects treated with omadacycline or vancomycin
Source: Antimicrob Agents Chemother. 2025 Jan 17;69(2):e01251-24. doi: 10.1128/aac.01251-24 (PMC11823362; doi:10.1128/aac.01251-24)
Supplement: Table S1 — Short-chain fatty acid proportional change from baseline in subjects given omadacycline compared to vancomycin. [file aac.01251-24-s0002.docx]

**Supplemental Table 1. Short-Chain Fatty Acid proportional change from baseline in subjects given omadacycline compared to vancomycin**

|  | Formic acid | | Acetic acid | | Propionic acid | | Isobutyric acid | | Butyric acid | | Isovaleric acid | | Valeric acid | | Hexanoic acid | |
| --- | --- | --- | --- | --- | --- | --- | --- | --- | --- | --- | --- | --- | --- | --- | --- | --- |
| **D** | OMC | VAN | OMC | VAN | OMC | VAN | OMC | VAN | OMC | VAN | OMC | VAN | OMC | VAN | OMC | VAN |
| **0** | 0 | 0 | 0 | 0 | 0 | 0 | 0 | 0 | 0 | 0 | 0 | 0 | 0 | 0 | 0 | 0 |
| **1** | 0.21 ± 0.73 | 0.13 ± 1.2 | 0 ± 1.01 | -0.22 ± 0.34 | -0.03±1 | -0.14 ± 0.38 | 3.02 ± 6.78 | -0.46 ± 0.5 | 0.14 ± 0.97 | -0.02 ± 1.51 | 4.6 ± 10.43 | 9.88 ± 18.38 | 1.22 ± 2.94 | 2.35 ± 3.19 | 1.48 ± 2.44 | 22.76 ± 57.65 |
| **2** | -0.27 ± 0.98 | -0.33 ± 0.93 | -0.66 ± 0.38 | -0.67 ± 0.32 | -0.4 ± 0.69 | -0.67 ± 0.27 | 0.12 ± 1.51 | -0.86 ± 0.26 | -0.9 ± 0.13 | -0.86 ± 0.24 | 4.34 ± 9.85 | 4.29 ± 6.53 | 1.76 ± 6.87 | 2.77 ± 6.37 | -0.8 ± 0.25 | 11.03 ± 29.04 |
| **3** | -0.28 ± 0.68 | -0.09 ± 1.49 | -0.59 ± 0.46 | -0.84 ± 0.24 | -0.22 ± 1.01 | -0.85 ± 0.18 | -0.25 ± 0.75 | -0.95 ± 0.06 | -0.42 ± 1.52 | -0.97 ± 0.03 | -0.18 ± 0.76 | -0.92 ± 0.1 | -0.69 ± 0.75 | -0.98 ± 0.05 | -0.22 ± 1.92 | -0.95 ± 0.07 |
| **4** | -0.68 ± 0.26 | -0.75 ± 0.15 | -0.79 ± 0.15 | -0.8 ± 0.15 | -0.69 ± 0.26 | -0.75 ± 0.18 | -0.3 ± 0.65 | -0.65 ± 0.57 | -0.98 ± 0.03 | -0.5 ± 1.2 | -0.22 ± 0.7 | 2.33 ± 4.82 | -0.98 ± 0.02 | -0.92 ± 0.13 | -0.89 ± 0.21 | -0.95 ± 0.11 |
| **5** | -0.63 ± 0.32 | 1.01 ± 4.65 | -0.79 ± 0.33 | -0.58 ± 0.46 | -0.81 ± 0.22 | -0.71 ± 0.22 | -0.54 ± 0.3 | -0.89 ± 0.15 | -1 ± 0.01 | -0.82 ± 0.41 | -0.55 ± 0.34 | 1.26 ± 3.59 | -0.99 ± 0.02 | -0.91 ± 0.16 | -0.95 ± 0.09 | 15.31 ± 41.3 |
| **6** | -0.75 ± 0.18 | -0.77 ± 0.15 | -0.69 ± 0.31 | -0.92 ± 0.08 | -0.51 ± 0.62 | -0.89 ± 0.06 | -0.48 ± 0.46 | -0.89 ± 0.25 | -0.96 ± 0.08 | -0.62 ± 0.9 | -0.44 ± 0.39 | -0.05 ± 1.31 | -0.96 ± 0.08 | -0.92 ± 0.17 | -0.92 ± 0.11 | -1 ± 0.01 |
| **7** | -0.74 ± 0.18 | -0.75 ± 0.27 | -0.68 ± 0.27 | -0.96 ± 0.05 | -0.39 ± 0.62 | -0.95 ± 0.03 | -0.26 ± 0.56 | -0.86 ± 0.24 | -0.94 ± 0.07 | -0.46 ± 1.49 | -0.29 ± 0.62 | -0.93 ± 0.1 | -0.87 ± 0.22 | -1 ± 0.01 | -0.89 ± 0.09 | -0.82 ± 0.44 |
| **9** | -0.75 ± 0.19 | 0.21 ± 2.52 | -0.62 ± 0.17 | -0.92 ± 0.08 | -0.46 ± 0.21 | -0.86 ± 0.12 | -0.19 ± 0.51 | -0.86 ± 0.26 | -0.94 ± 0.07 | -0.53 ± 1.14 | -0.18 ± 0.51 | -0.34 ± 0.71 | -0.89 ± 0.2 | -0.98 ± 0.02 | -0.93 ± 0.09 | -1 ± 0.01 |
| **10** | -0.8 ± 0.13 | -0.62 ± 0.66 | -0.7 ± 0.27 | -0.84 ± 0.27 | -0.51 ± 0.57 | -0.85 ± 0.17 | -0.17 ± 0.57 | -0.84 ± 0.26 | -0.96 ± 0.06 | -0.21 ± 2.19 | -0.2 ± 0.63 | -0.86 ± 0.11 | -0.85 ± 0.4 | -1 ± 0 | -0.93 ± 0.09 | -0.89 ± 0.25 |

D: Day of therapy; OMC: omadacycline; VAN: vancomycin
